# Comparison of the tumor immune microenvironment of primary hormone receptor-negative HER2-positive and triple negative breast cancer

**DOI:** 10.1038/s41523-021-00332-7

**Published:** 2021-09-23

**Authors:** Hanne Vos, Kathleen Lambein, François Richard, Bram Mariën, Ines Nevelsteen, Kevin Punie, Hans Wildiers, Lieze Berben, Annouschka Laenen, Giuseppe Floris, Christine Desmedt, Ann Smeets

**Affiliations:** 1 Department of Surgical Oncology, University Hospitals Leuven; Department of Oncology, KU Leuven, Leuven, Belgium; 2 Laboratory for Translational Breast Cancer Research, Department of Oncology, KU Leuven, Leuven, Belgium; 3 Multidisciplinary Breast Centre, University Hospitals Leuven, Leuven, Belgium; 4 Laboratory of Experimental Oncology, Department of General Medical Oncology, University Hospitals Leuven, KU Leuven, Leuven, Belgium; 5 Leuven Biostatistics and Statistical Bioinformatics Centre (L-BioStat), KU Leuven, Leuven, Belgium; 6 Laboratory of Translational Cell & Tissue Research, Department of Imaging and Pathology, University Hospitals Leuven, KU Leuven, Leuven, Belgium

**Keywords:** Breast cancer, Immunoediting

## Abstract

The vast majority of studies investigating immune checkpoint inhibition (ICI) in patients with breast cancer have focused on triple-negative breast cancer (TNBC). In this study, we compared the tumor immune microenvironment (TIME) between TNBC and hormone receptor-negative HER2-positive breast cancer based on a selection of immune markers at the protein level in an institutional retrospective series. Additionally, we performed a similar comparison using publicly available transcriptomics data. Altogether, the results show a comparable TIME in both groups, with possible implications for the use of ICI in patients with hormone receptor-negative HER2-positive breast tumors.

Research on immune checkpoint inhibitors (ICI) in breast cancer (BC) has mainly focused on triple-negative breast cancer (TNBC), due to the immunogenicity of these tumors and the lack of targeted therapies to treat these patients. Although the prognosis of patients with primary HER2-positive BC has significantly increased since the introduction of anti-HER2-targeted therapies, the vast majority of patients eventually develops resistance, leading to disease relapse and progression^1^. Currently, the 5-year relative survival percentages for HER2-positive BC and TNBC are 84 and 76.9%, respectively^2^.

ICI might therefore represent an additional treatment option for these patients, as recently suggested by the PANACEA^3^ and KATE2^4^ trials in the advanced setting. We further refer to the review from Costa and Czerniecki for a more extensive review of the ongoing trials^5^. So far, the tumor immune microenvironment (TIME) of HER2-positive BC remains poorly characterized and existing data usually comprise pooled analyses of hormone receptor (HR)-positive and HR-negative HER2-positive tumors^6^. Moreover, most research on the TIME in HER2-positive BC has so far focused on the prognostic effect of stromal tumor infiltrating lymphocytes (sTIL)^7,8^, and the predictive value for response to neo-adjuvant chemotherapy or HER2-targeted therapy^3,9–12^.

In this study, we therefore aimed to compare the TIME of TNBC and HER2-positive HR-negative non-special type BC tumors, in order to explore potential similarities or differences.

In the first part of our study, we analyzed sTIL, scored according to standard guidelines^13^, and multiple markers (CD3, CD4, CD8, CD68, CD73, PD-1, PD-L1, FOXP3, and Ki67) by immunohistochemistry (IHC) on formalin-fixed paraffin-embedded samples from tumor resection specimens of 163 patients with HR-negative stage 2–3 BC. All patients underwent upfront surgery at UZ Leuven (Leuven, Belgium) between the 1st of January 2005 and the 31st of December 2010. Tumors from 110 (67%) and 53 (33%) patients were triple-negative and HR-negative/HER2-positive, respectively. When comparing the clinico-pathological characteristics of patients with TNBC and HER2-positive BC, we observed, as expected^14,15^, a higher proportion of axillary lymph node metastases at diagnosis in patients with HER2-positive tumors compared to patients with TNBC (Supplementary Table 1).

Considering standardly acknowledged cutpoints of sTIL for defining highly infiltrated tumors (30 and 60%)^7,8^, we observed 28 and 11%, respectively, highly infiltrated TNBC tumors and 21% and 6% highly infiltrated HER2-positive tumors. Using a cutpoint of ≥1%, we found 94% PD-1 positive and 17% PD-L1 positive TNBC tumors, and 93% PD-1 and 19% PD-L1 positive tumors. A comparative analysis of TNBC and HER2-positive BC revealed no significant differences regarding sTIL and the majority of the investigated immune cell subpopulations. We nevertheless identified significantly higher levels of CD68^+^ (*p* = 0.002) and FOXP3^+^ cells (*p* = 0.005) in TNBC as compared to HER2-positive tumors (Fig. 1A). Given the higher proportion of axillary lymph node involvement in patients with HER2-positive BC, we performed Firth logistic regressions with nodal status as covariate and confirmed the associations independently of the nodal status (Supplementary Fig. 1 and Supplementary Table 2).

**Fig. 1:**
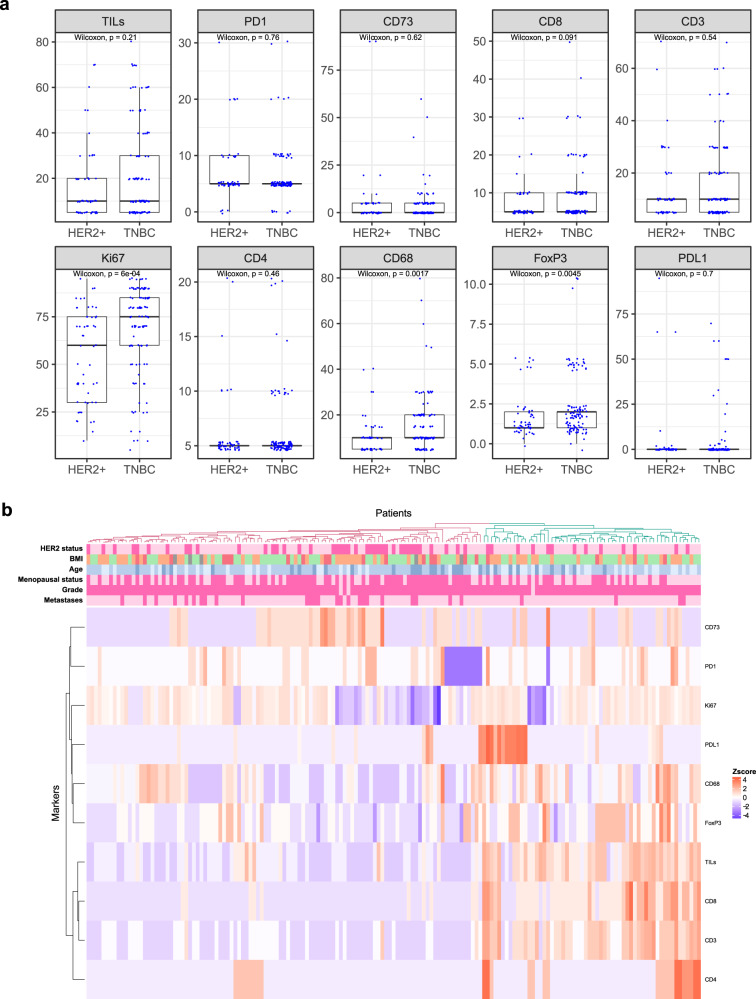
Characteristics of the 163 tumor samples, 110 HER2-/ER-/PR- and 53 HER2+/ER-/PR-. **A** Immune markers scored by an experienced pathologist. Wilcoxon test *p*-values are indicated for each marker. The center line is the median, the bounds of box are the first and third quartile and the whiskers have a length of 1.5 interquartile range. The dots represent the actual individual data. **B** Heatmap of the hierarchical clustering. The 2 clusters obtained are depicted in red and green on the patient dendrogram, so called low and high immune infiltrated respectively, they account for 105 and 58 patients respectively and present 38% (40/105) and 22% (13/58) HER2+ patients respectively (fisher *p*-value: 0.05432). Annotations are given for HER2 status (negative or positive in light and dark pink respectively); BMI ([18–25], [25–30],[30–42], in green, orange, red respectively; age ([27–45], [45–70], [70–90], from light to dark blue respectively); menopausal status (negative or positive in light and dark pink respectively); grade (II or III in light and dark pink respectively); metastases (absent or present in light and dark pink respectively). Missing values appear in gray.

We further generated an unsupervised hierarchical clustering with the various immune scores (Fig. 1B). The tumors were distributed in two main clusters. The smallest cluster of 58 patients was characterized by infiltrated tumors, i.e. higher sTIL levels and higher expression of CD3 and CD8. We also observed a subcluster of tumors with high PD-L1 expression. The second main cluster of 105 patients was generally characterized by low sTIL levels and low CD3 and CD8 expression. We observed a subcluster with CD73-positive tumors and another with PD-1 expression.

We then explored whether the standard clinico-pathological characteristics correlated with the expression of the selected immune parameters within TNBC and HER2-positive tumors separately.

Focusing on patients with TNBC, we observed that older patients had significantly less sTIL (*ρ* = −0.250, *p* = 0.008) and lower expression of CD3 (Supplementary Table 3). The same result towards lower expression was observed in postmenopausal patients for sTIL, CD3, and CD8 (Supplementary Table 4). Body mass index did not correlate with sTIL (Supplementary Table 3), but patients with a high BMI had a significant higher expression of CD73 (*ρ* = 0.202, *p* = 0.036). No associations between immune parameters and tumor size were found (Supplementary Table 5). However an association was noted between CD73 and lymph node involvement. Patients with a positive pathological nodal status had a significant higher expression of CD73 compared to patients with a negative nodal status (Supplementary Table 6).

In the group of patients with a HER2-positive tumor, no significant associations between the immune parameters and standard clinico-pathological characteristics were found (Supplementary tables 3–6).

To further characterize the TIME of HR-negative tumors according to HER2 status, we analyzed the transcriptomic data from METABRIC, which was available for 320 TNBC and 134 HR-negative HER2-positive tumors. Consistently with what we observed in our institutional cohort, we observed a larger proportion of patients with HER2-positive BC having axillary lymph node involvement at diagnosis compared to patients with TNBC (Supplementary Table 5). The CIBERSORT deconvolution^16^ was available for 265 TNBC-, and 109 HR-negative HER2-positive tumors. The analyses showed significant differences between the two groups concerning plasma cells (*p* = 0.014), memory resting CD4+ T-cells (*p* < 0.001) and resting mast cells (*p* < 0.001), with a consistent higher expression in HER2-positive BC (Fig. 2). No significant difference was observed for all other immune cell subpopulations.

**Fig. 2:**
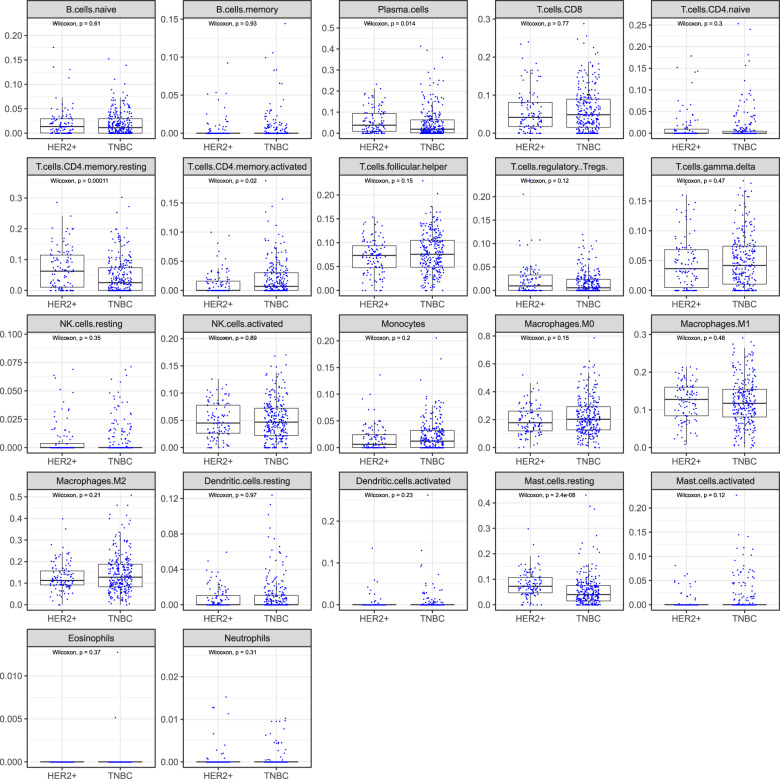
CIBERSORT deconvolution for 265 TNBC, and 109 HER2+ /ER-/PR- patients. Wilcoxon test *p*-values are indicated for each cell types.

Acknowledging the relatively small sample size, we currently showed that the TIME is very similar between HR-negative HER2-positive tumors and TNBC, using central pathology assessment of various markers. However, at the immunohistochemical level, we observed small differences in our study population in the levels of CD68^+^ and FOXP3^+^ cells in TNBC as compared to HER2-positive tumors. These results were not confirmed at the transcriptomic level in the METABRIC dataset, where higher levels of plasma cells, memory resting CD4+ T-cells, and resting mast cells were observed in HR-negative/HER2-positive tumors as compared to TNBC. These differences could be explained by the differences in study population or most probably by the differences between the transcriptomic and protein level, as recently documented by Nederlof and colleagues^17^. Of note, the observed high levels of sTIL and PD-L1-positivity rate for a considerable proportion of HER2-positive tumors are in agreement with previous research^9,10,13,18^.

The similarity in the TIME for HER2-positive and TNBC tumors suggests that patients with HER2-positive BC could also benefit from immunotherapy combined with established or novel HER2-targeted therapy, e.g. trastuzumab emtansine (T-DM1). The potential benefit of ICI in patients with metastatic HER2+ BC has been reported in several studies and PD-L1 positivity and sTIL seemed to be predictive^5^. No results are available yet for early BC, with a few trials that are currently ongoing such as a phase 2 trial with pembrolizumab (NCT 03747120) and a phase 3 trial with atezolizumab (NCT 03726879).

To conclude, our characterization of the TIME in HER2-positive BC suggests a potential role for immunotherapy in these patients. These findings warrant more research on combining immunotherapy with traditional or novel anti-HER2 agents, which could lead to more tailored options for patients with early HER2-positive BC.

## Methods

### Patients

A consecutive series of female patients with a non-special type ER/PR negative invasive adenocarcinoma of at least 2 cm diameter were included. All patients underwent upfront surgery at the University Hospitals Leuven (UHL) between 2005 and 2010. Patients were retrospectively selected from the prospectively collected database of the Multidisciplinary Breast Center at the UHL. An informed consent was obtained for all patients in this database. Receptor status was defined according to ASCO/CAP guidelines^19,20^. Patients who received neo-adjuvant systemic treatment were excluded. Clinico-pathological parameters of all patients were retrieved from the hospital information system. BMI categories were defined as lean (<25 kg/m^2^) versus overweight (≥25 and <30 kg/m^2^) and obese (≥30 kg/m^2^). The study protocol was approved by the local medical ethics committee of the UHL (S58910, 9 May 2016).

### H&E and immunohistochemistry

Archival hematoxylin and eosin (H&E) stained slides were reviewed by an experienced pathologist (K.L.). sTIL were evaluated according to the guidelines of the International Immuno-Oncology Biomarkers Working Group^13^. Paraffin blocks of the primary site of the BC tumor were selected based on the pathology report for diagnostics and H&E stained slides. HER2 expression was evaluated by means of immunohistochemistry, according to the Guidelines of the American Society of Clinical Oncology/College of American Pathologists (ASCO/CAP)^21^. Equivocal cases (score 2+) received additional testing by FISH in order to detect HER2 amplification. Score 3+ cases received FISH confirmation as well, as this is mandatory for reimbursement of HER2-targeted therapy in Belgium.

Immunohistochemical stains were performed on 5 μm thick sections using an automatic immunostainer (Bond Max Autostainer, Leica) according to the manufacturer’s instructions.

A polyclonal CD3 antibody (ready-to-use, IR50361, Agilent) and monoclonal antibodies were used for CD4 (clone 4B12, ready-to-use, IR64961, Agilent), CD8 (clone C8/144B, ready-to-use, IR62361, Agilent), CD68 (clone KP1, ready-to-use, IR60961, Agilent), CD73 (clone 1D7, 1/200 dilution, ab133582, Abcam), FoxP3 (clone 22510, 1/200 dilution, ab22510, Abcam), Ki67 (clone MIB-1, ready-to-use, IR62661, Agilent), and PD1 (clone NAT105, 1/100 dilution, ab52587, Abcam). CD3, CD4, CD8, FOXP3, and PD1 expression on sTIL and CD73 expression on tumoral cells were assessed semi quantitatively. PD-L1 (22C3 antibody, 2 μg/mL, Dako-Agilent) immunohistochemistry was performed by Qualtek Molecular Laboratories (Newtown PA, USA) as per agreement with Merck. QualTek provided a modified proportion score (MPS) indicating the proportion of PD-L1-expressing tumor cells and mononuclear inflammatory cells within tumor nests.

### Statistical analyses

The analysis of continuous variables was performed using the Mann–Whitney *U* test for comparing two groups, or the Kruskal–Wallis test for more than two groups. The association among categorical variables was assessed by Fisher exact test and association among continuous variables was estimated by means of the Spearman correlation coefficient. Results were presented as hazard ratios with 95% confidence intervals. All tests were two-sided, while a 5% significance level was adopted. No correction for multiplicity was performed. Data analysis was conducted by an expert biostatistician with SAS software (version 9.4 of the SAS System for Windows).

### Bioinformatics analyses

Wilcoxon tests were performed to compare continuous to categorical variables. *P*-values were two-sided and statistical significance considered for *p* < 0.05. Association between IHC markers taken as continuous percentage and subtypes were assessed by Firth logistic regressions, considering subtypes as the outcome variable (HER2-positive vs TNBC) and adjusting for nodal status (positive vs negative) in the multivariable models. All analyses were performed using R 3.5.2.

Unsupervised hierarchical clustering of the continuous markers scored on tumor tissues was performed. Data were scaled and log transformed prior to the z-score computation. Clustering was based on Euclidian distance and the ward linkage criterion. Optimal cluster count was established by the gap statistic method^22^ and evaluated to 2.

The METABRIC dataset^23^, including clinical data and normalized gene expression, was retrieved through cBioportal^24^. Subtypes were assessed according to the HR and HER2 status. 320 TNBC and 134 HER2+HR-patients were retrieved.

METABRIC transcriptomic data were processed through CIBERSORT^16^ and EPIC^25^ software in their online versions. CIBERSORT were run using the default signature matrix, 1000 permutations, in absolute and relative mode and without quantile normalization as advised for RNA-Seq data. Only deconvolution solutions reaching significance were kept for downstream analysis given a dataset of 265 TNBC, and 109 HER2+HR- patients.

### Reporting summary

Further information on research design is available in the Nature Research Reporting Summary linked to this article.

## Supplementary information

Supplementary Information

Reporting Summary

## Data Availability

The METABRIC dataset analyzed during the current study is available in the cbioportal repository, https://www.cbioportal.org/study/summary?id=brca_metabric. The H&E and immunohistochemistry datasets generated and analyzed during the current study are not publicly available but will be made available upon reasonable request, following ethics committee approval and a data transfer agreement, to guarantee the General Data Protection Regulation.
