# Supplementary material for: Comparison of the tumor immune microenvironment of primary hormone receptor-negative HER2-positive and triple negative breast cancer
Source: NPJ Breast Cancer. 2021 Sep 23;7:128. doi: 10.1038/s41523-021-00332-7 (PMC8460670; doi:10.1038/s41523-021-00332-7)
Supplement: Supplementary file 1 — Supplementary Information [file 41523_2021_332_MOESM1_ESM.pdf]

**Supplementary Information**

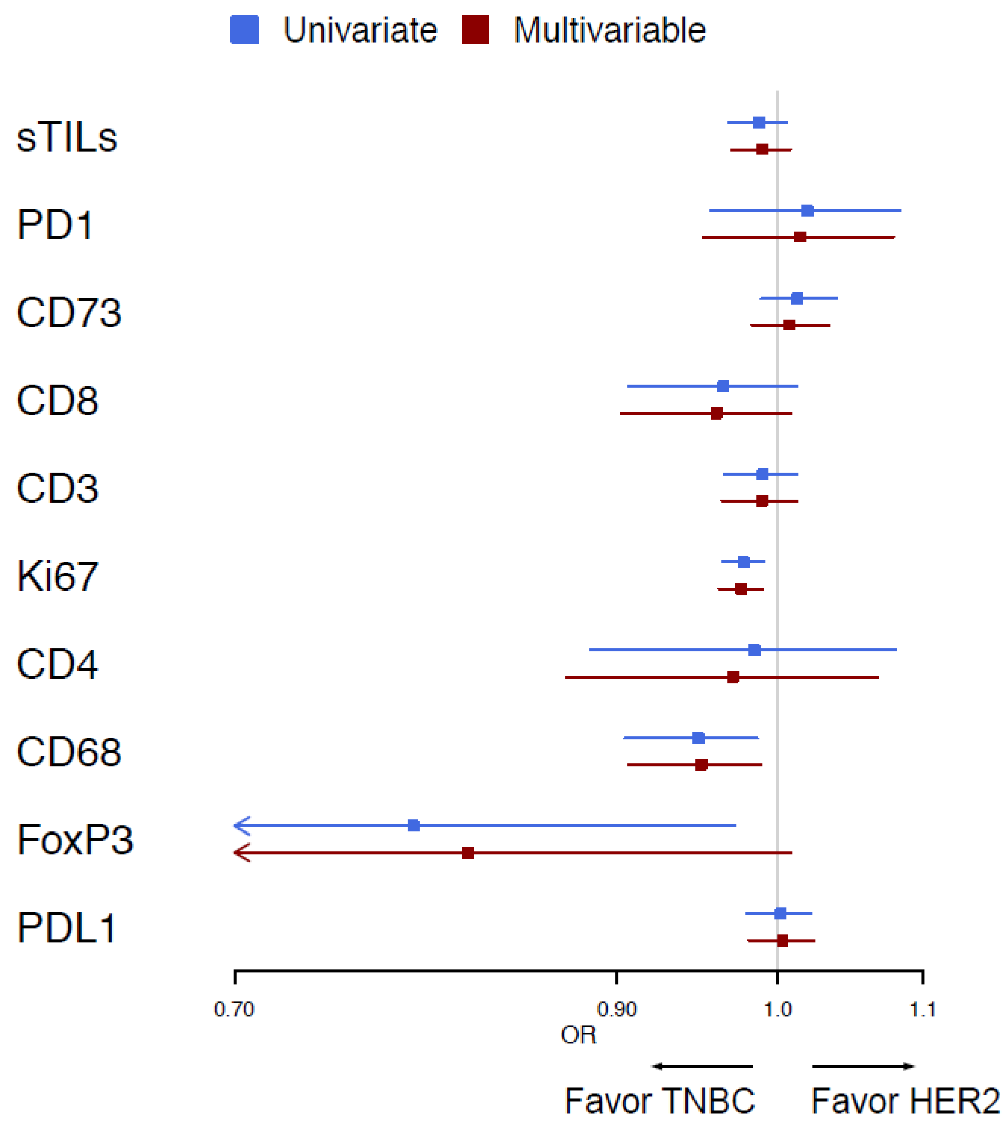


**Supplementary Figure 1**: Forest plot of the First logistic regressions to assess the association between subtypes and IHC markers taken as continuous percentage. Univariable model in blue, multivariable in red adjusted for nodal status (positive vs negative), odd ratios and confidence intervals are reported as squares and horizontal lines respectively.

**Supplementary Table 1**: Clinico pathological variable in the current cohort according the BC subtypes. P values in bold fall below the 0.05 significance level.

| | | HER2+ | TNBC | P-value |
|---|---|---|---|---|
| **N** | | **53** | **110** | |
| **Age (continuous)** | Median | 54 | 52 | 0.243 |
| | IQR | (47.0-69.0) | (43.0-65.8) | |
| | Mean | 56.7 | 54.0 | |
| | sd | 14.7 | 13.6 | |
| | Range | (27-87) | (29-86) | |
| **Age (category)** | ≤ 45 | 12 (22.6) | 35 (31.8) | 0.396 |
| | ≤ 70 | 29 (54.7) | 58 (52.7) | |
| | > 70 | 12 (22.6) | 17 (15.5) | |
| **Menopausal.status** | Pre-menopausal | 21 (39.6) | 51 (46.4) | 0.501 |
| | Post-menopausal | 32 (60.4) | 59 (53.6) | |
| **Tumor grade** | 1 | 0 (0.0) | 0 (0.0) | 0.033 |
| | 2 | 3 (5.7) | 0 (0.0) | |
| | 3 | 50 (94.3) | 110 (100.0) | |
| **pT** | 1 | 0 (0.0) | 0 (0.0) | 0.069 |
| | 2 | 45 (84.9) | 104 (94.5) | |
| | 3 | 8 (15.1) | 6 (5.5) | |
| **pN** | 0 | 18 (34.0) | 61 (55.5) | **0.001** |
| | 1 | 20 (37.7) | 43 (39.1) | |
| | 2 | 10 (18.9) | 3 (2.7) | |
| | 3 | 5 (9.4) | 3 (2.7) | |
| **Nodal status** | Negative | 18 (34.0) | 64 (58.2) | **0.005** |
| | Positive | 35 (66.0) | 46 (41.8) | |
| | | | | |
| **N** | | | **107** | |
| **BMI (continuous)** | Median | 25.2 | 24.8 | 0.549 |
| | IQR | (21.8-27.3) | (22.6-27.8) | |
| | Range | (18.9-34.4) | (18.8-42) | |
| | Mean | 25.0 | 25.7 | |
| | sd | 3.8 | 4.7 | |
| | Missing | 2 | 3 | |
| **BMI (category)** | Lean | 25 (49.0) | 56 (52.3) | 0.790 |
| | Overweight | 20 (39.2) | 36 (33.6) | |
| | Obese | 6 (11.8) | 15 (14.0) | |
| | Missing | 2 | 3 | |

**Supplementary Table 2**: Detail of the Firth logistic regressions represented in Supplementary Figure 1

| Variable | OR uni | CI95 uni lower | CI95 uni upper | P-values uni | OR multi | CI95 multi lower | CI95 multi upper | P-values Multi |
|---|---|---|---|---|---|---|---|---|
| sTILs | 0.988 | 0.968 | 1.005 | <0.001 | 0.990 | 0.970 | 1.008 | 0.284 |
| PD1 | 1.019 | 0.957 | 1.083 | 0.767 | 1.015 | 0.952 | 1.079 | 0.644 |
| CD73 | 1.013 | 0.989 | 1.040 | 0.250 | 1.018 | 0.983 | 1.034 | 0.530 |
| CD8 | 0.965 | 0.907 | 1.013 | <0.001 | 0.960 | 0.902 | 1.009 | 0.114 |
| CD3 | 0.990 | 0.965 | 1.013 | 0.767 | 0.996 | 0.964 | 1.013 | 0.395 |
| Ki67 | 0.978 | 0.964 | 0.991 | 0.250 | 0.976 | 0.962 | 0.990 | <0.001 |
| CD4 | 0.984 | 0.884 | 1.080 | <0.001 | 0.988 | 0.871 | 1.068 | 0.556 |
| CD68 | 0.949 | 0.904 | 0.987 | 0.767 | 0.940 | 0.906 | 0.989 | 0.009 |
| FoxP3 | 0.787 | 0.611 | 0.973 | 0.250 | 0.781 | 0.633 | 1.009 | 0.061 |
| PDL1 | 1.002 | 0.980 | 1.022 | <0.001 | 1.008 | 0.981 | 1.024 | 0.763 |

**Supplementary Table 3**: Association of Age and BMI with different immune parameters according the BC subtypes. P values in bold fall below the 0.05 significance level.

| TNBC | | | |
|---|---|---|---|
| **Association of Age with (N = 110)** | **ρ** | **95% Confidence Interval** | **P-value** |
| TILs | -0.250 | (-0.417; -0.065) | **0.008** |
| PD1 | -0.034 | (-0.220; 0.155) | 0.726 |
| CD73 | 0.028 | (-0.160; 0.214) | 0.770 |
| CD8 | -0.154 | (-0.331; 0.035) | 0.107 |
| CD3 | -0.241 | (-0.409; -0.055) | **0.011** |
| Ki67 | -0.286 | (-0.448; -0.103) | **0.002** |
| CD4 | -0.145 | (-0.323; 0.044) | 0.131 |
| CD68 | -0.033 | (-0.218; 0.156) | 0.736 |
| FoxP3 | -0.130 | (-0.309; 0.060) | 0.178 |
| PDL1 | -0.079 | (-0.262; 0.110) | 0.411 |

| HER2+ | | | |
|---|---|---|---|
| **Association of Age with (N = 53)** | **ρ** | **95% Confidence Interval** | **P-value** |
| TILs | -0.191 | (-0.437; 0.086) | 0.172 |
| PD1 | -0.090 | (-0.351; 0.186) | 0.524 |
| CD73 | -0.228 | (-0.467; 0.048) | 0.101 |
| CD8 | -0.115 | (-0.372; 0.162) | 0.415 |
| CD3 | -0.244 | (-0.480; 0.031) | 0.079 |
| Ki67 | -0.227 | (-0.467; 0.048) | 0.102 |
| CD4 | -0.189 | (-0.435; 0.088) | 0.177 |
| CD68 | -0.106 | (-0.365; 0.170) | 0.452 |
| FoxP3 | -0.134 | (-0.389; 0.142) | 0.339 |
| PDL1 | 0.087 | (-0.188; 0.349) | 0.536 |

| TNBC | | | |
|---|---|---|---|
| **Association of BMI with (N = 110)** | **ρ** | **95% Confidence Interval** | **P-value** |
| TILs | -0.190 | (-0.366; 0.001) | 0.050 |
| PD1 | 0.144 | (-0.048; 0.324) | 0.139 |
| CD73 | 0.202 | ( 0.012; 0.377) | **0.036** |
| CD8 | -0.180 | (-0.357; 0.011) | 0.063 |
| CD3 | -0.174 | (-0.351; 0.018) | 0.074 |
| Ki67 | 0.089 | (-0.103; 0.274) | 0.362 |
| CD4 | -0.053 | (-0.240; 0.138) | 0.589 |
| CD68 | 0.163 | (-0.029; 0.341) | 0.094 |
| FoxP3 | -0.000 | (-0.190; 0.189) | 0.997 |
| PDL1 | -0.099 | (-0.283; 0.093) | 0.312 |

| HER2+ | | | |
|---|---|---|---|
| **Association of BMI with (N = 53)** | **ρ** | **95% Confidence Interval** | **P-value** |
| TILs | 0.204 | (-0.078; 0.452) | 0.152 |
| PD1 | -0.205 | (-0.453; 0.077) | 0.150 |
| CD73 | -0.248 | (-0.488; 0.032) | 0.080 |
| CD8 | 0.201 | (-0.081; 0.450) | 0.157 |
| CD3 | 0.135 | (-0.148; 0.395) | 0.348 |
| Ki67 | -0.268 | (-0.504; 0.011) | 0.057 |
| CD4 | 0.129 | (-0.153; 0.389) | 0.370 |
| CD68 | 0.026 | (-0.252; 0.299) | 0.857 |
| FoxP3 | -0.020 | (-0.293; 0.258) | 0.892 |
| PDL1 | 0.110 | (-0.172; 0.373) | 0.444 |

**Supplementary Table 4**: Association of menopausal status with different immune parameters according the BC subtypes. P values in bold fall below the 0.05 significance level.

| | | TNBC | | | HER2+ | | |
|---|---|---|---|---|---|---|---|
| **Variable** | **Statistic** | **Non menopausal (N=51)** | **Menopausal (N=59)** | **P-value** | **Non menopausal (N=21)** | **Menopausal (N=32)** | **P-value** |
| **TILs** | Mean | 25.9 | 17.0 | **0.029** | 16.9 | 16.6 | 0.354 |
| | Std | 22.5 | 16.6 | | 15.5 | 17.7 | |
| | Median | 20.0 | 10.0 | | 10.0 | 10.0 | |
| | IQR | (10.0; 40.0) | (5.0; 20.0) | | (10.0; 20.0) | (5.0; 20.0) | |
| | Range | (5.0; 80.0) | (5.0; 60.0) | | (5.0; 70.0) | (5.0; 70.0) | |
| **PD1** | Mean | 6.8 | 6.4 | 0.664 | 7.4 | 6.8 | 0.393 |
| | Std | 5.0 | 4.7 | | 4.6 | 6.2 | |
| | Median | 5.0 | 5.0 | | 5.0 | 5.0 | |
| | IQR | (5.0; 10.0) | (5.0; 5.0) | | (5.0; 10.0) | (5.0; 7.5) | |
| | Range | (0.0; 30.0) | (0.0; 30.0) | | (5.0; 20.0) | (0.0; 30.0) | |
| **CD73** | Mean | 3.1 | 4.6 | 0.396 | 8.3 | 4.7 | 0.200 |
| | Std | 6.7 | 10.4 | | 19.6 | 15.8 | |
| | Median | 0.0 | 0.0 | | 5.0 | 0.0 | |
| | IQR | (0.0; 5.0) | (0.0; 5.0) | | (0.0; 5.0) | (0.0; 5.0) | |
| | Range | (0.0; 40.0) | (0.0; 60.0) | | (0.0; 90.0) | (0.0; 90.0) | |
| **CD8** | Mean | 11.7 | 7.5 | **0.005** | 8.8 | 6.9 | 0.376 |
| | Std | 9.8 | 4.9 | | 6.9 | 4.7 | |
| | Median | 10.0 | 5.0 | | 5.0 | 5.0 | |
| | IQR | (5.0; 10.0) | (5.0; 10.0) | | (5.0; 10.0) | (5.00; 7.50) | |
| | Range | (5.0; 50.0) | (5.0; 30.0) | | (5.0; 30.0) | (5.0; 30.0) | |
| **CD3** | Mean | 19.1 | 12.4 | **0.006** | 16.2 | 11.4 | 0.227 |
| | Std | 16.9 | 12.4 | | 15.6 | 11.8 | |
| | Median | 10.0 | 5.0 | | 10.0 | 10.0 | |
| | IQR | (5.0; 30.0) | (5.0; 10.0) | | (5.0; 20.0) | (5.0; 10.0) | |
| | Range | (5.0; 70.00) | (5.0; 60.0) | | (5.0; 70.0) | (5.0; 60.0) | |
| **Ki67** | Mean | 75.6 | 61.4 | **0.003** | 61.9 | 49.8 | 0.121 |
| | Std | 16.8 | 25.5 | | 23.1 | 23.8 | |
| | Median | 80.0 | 70.0 | | 70.0 | 42.5 | |
| | IQR | (70.0; 90.0) | (40.0; 85.0) | | (40.0; 75.0) | (30.0; 72.5) | |
| | Range | (15.0; 95.0) | (5.0; 95.0) | | (20.0; 95.0) | (10.0; 90.0) | |
| **CD4** | Mean | 7.2 | 5.7 | 0.121 | 6.7 | 5.8 | 0.315 |
| | Std | 4.5 | 1.7 | | 4.0 | 2.9 | |
| | Median | 5.0 | 5.0 | | 5.0 | 5.0 | |
| | IQR | (5.0; 5.0) | (5.0; 5.0) | | (5.0; 5.0) | (5.0; 5.0) | |
| | Range | (5.0; 20.0) | (5.0; 10.0) | | (5.0; 20.0) | (5.0; 20.0) | |
| **CD68** | Mean | 17.0 | 15.2 | 0.641 | 13.3 | 9.4 | 0.053 |
| | Std | 13.7 | 12.2 | | 9.4 | 6.9 | |
| | Median | 10.0 | 10.0 | | 10.0 | 7.5 | |
| | IQR | (10.0; 20.0) | (10.0; 20.0) | | (10.0; 15.0) | (5.0; 10.0) | |
| | Range | (5.0; 70.0) | (5.0; 80.0) | | (5.0; 40.0) | (5.0; 40.0) | |
| **FoxP3** | Mean | 2.4 | 2.3 | 0.325 | 1.7 | 1.7 | 0.430 |
| | Std | 1.9 | 2.0 | | 1.2 | 1.51 | |
| | Median | 2.0 | 2.0 | | 1.0 | 1.0 | |
| | IQR | (1.0; 2.0) | (1.0; 2.0) | | (1.0; 2.0) | (1.0; 2.0) | |
| | Range | (0.0; 10.0) | (0.0; 10.0) | | (1.0; 5.0) | (0.0; 5.0) | |
| **PDL1** | Mean | 5.6 | 3.3 | 0.806 | 3.3 | 5.3 | 0.589 |
| | Std | 16.3 | 11.2 | | 14.1 | 20.0 | |
| | Median | 0.0 | 0.0 | | 0.0 | 0.0 | |
| | IQR | (0.0; 0.0) | (0.0; 0.0) | | (0.0; 0.0) | (0.0; 0.0) | |
| | Range | (0.0; 70.0) | (0.0; 60.0) | | (0.0; 65.0) | (0.0; 95.0) | |

**Supplementary Table 5**: Association of tumorsize with different immune parameters according the BC subtypes. P values in bold fall below the 0.05 significance level.

| | | TNBC | | | HER2+ | | |
|---|---|---|---|---|---|---|---|
| **Variable** | **Statistic** | **pT2 (N=104)** | **pT3-4 (N=6)** | **P-value** | **pT2 (N=45)** | **pT3-4 (N=8)** | **P-value** |
| **TILs** | Mean | 21.1 | 21.7 | 0.829 | 16.9 | 15.6 | 0.552 |
| | Std | 20.0 | 20.2 | | 15.9 | 22.1 | |
| | Median | 10.0 | 20.0 | | 10.0 | 10.0 | |
| | IQR | (5.0; 30.0) | (5.0; 20.0) | | (5.0; 20.0) | (5.0; 10.0) | |
| | Range | (5.0; 80.0) | (5.0; 60.0) | | (5.0; 70.0) | (5.0; 70.0) | |
| **PD1** | Mean | 6.6 | 5.8 | 0.896 | 7.6 | 4.4 | 0.081 |
| | Std | 4.9 | 2.0 | | 5.9 | 1.8 | |
| | Median | 5.0 | 5.0 | | 5.0 | 5.0 | |
| | IQR | (5.0; 5.0) | (5.0; 5.0) | | (5.0; 10.0) | (5.0; 5.0) | |
| | Range | (0.0; 30.0) | (5.0; 10.0) | | (0.0; 30.0) | (0.0; 5.0) | |
| **CD73** | Mean | 3.7 | 7.5 | 0.945 | 6.1 | 6.2 | 0.051 |
| | Std | 8.4 | 16.0 | | 18.7 | 6.4 | |
| | Median | 0.0 | 0.0 | | 0.0 | 5.0 | |
| | IQR | (0.0; 5.0) | (0.0; 5.0) | | (0.0; 5.0) | (2.5; 7.5) | |
| | Range | (0.0; 60.0) | (0.0; 40.0) | | (0.0; 90.0) | (0.0; 20.0) | |
| **CD8** | Mean | 9.6 | 6.7 | 0.508 | 7.9 | 6.2 | 0.718 |
| | Std | 8.0 | 2.6 | | 6.1 | 2.3 | |
| | Median | 5.0 | 5.0 | | 5.0 | 5.0 | |
| | IQR | (5.0; 10.0) | (5.0; 10.0) | | (5.0; 10.0) | (5.0; 7.5) | |
| | Range | (5.0; 50.0) | (5.0; 10.0) | | (5.0; 30.0) | (5.0; 10.0) | |
| **CD3** | Mean | 16.0 | 7.5 | 0.068 | 14.1 | 8.7 | 0.451 |
| | Std | 15.2 | 6.1 | | 14.4 | 5.2 | |
| | Median | 10.0 | 5.0 | | 10.0 | 7.50 | |
| | IQR | (5.0; 20.0) | (5.0; 5.0) | | (5.0; 20.0) | (5.0; 10.0) | |
| | Range | (5.0; 70.0) | (5.0; 20.0) | | (5.0; 70.0) | (5.0; 20.0) | |
| **Ki67** | Mean | 67.6 | 74.2 | 0.324 | 54.6 | 55.0 | 1.000 |
| | Std | 22.9 | 24.6 | | 24.8 | 20.9 | |
| | Median | 72.5 | 80.0 | | 60.0 | 57.5 | |
| | IQR | (60.0; 85.0) | (80.0; 90.0) | | (30.0; 75.0) | (40.0; 72.5) | |
| | Range | (5.0; 95.0) | (25.0; 90.0) | | (10.0; 95.0) | (20.0; 80.0) | |
| **CD4** | Mean | 6.3 | 6.7 | 0.393 | 6.3 | 5.0 | 0.245 |
| | Std | 3.4 | 2.6 | | 3.6 | 0.0 | |
| | Median | 5.0 | 5.0 | | 5.0 | 5.0 | |
| | IQR | (5.0; 5.0) | (5.0; 10.0) | | (5.0; 5.0) | (5.0; 5.0) | |
| | Range | (5.0; 20.0) | (5.0; 10.0) | | (5.0; 20.0) | (5.0; 5.0) | |
| **CD68** | Mean | 16.2 | 13.3 | 0.844 | 11.3 | 8.7 | 0.702 |
| | Std | 13.1 | 6.8 | | 8.7 | 3.5 | |
| | Median | 10.0 | 15.0 | | 10.0 | 10.0 | |
| | IQR | (10.0; 20.0) | (5.0; 20.0) | | (5.0; 15.0) | (5.0; 10.0) | |
| | Range | (5.0; 80.0) | (5.0; 20.0) | | (5.0; 40.0) | (5.0; 15.0) | |
| **FoxP3** | Mean | 2.4 | 1.3 | 0.119 | 1.7 | 1.6 | 0.239 |
| | Std | 2.0 | 0.5 | | 1.5 | 0.5 | |
| | Median | 2.0 | 1.0 | | 1.0 | 2.0 | |
| | IQR | (1.0; 2.0) | (1.0; 2.0) | | (1.0; 2.0) | (1.0; 2.0) | |
| | Range | (0.0; 10.0) | (1.0; 2.0) | | (0.0; 5.0) | (1.0; 2.0) | |
| **PDL1** | Mean | 4.6 | 0.5 | 0.905 | 5.1 | 1.7 | 0.070 |
| | Std | 14.1 | 1.2 | | 19.3 | 3.4 | |
| | Median | 0.0 | 0.0 | | 0.0 | 0.0 | |
| | IQR | (0.0; 0.0) | (0.0; 0.0) | | (0.0; 0.0) | (0.0; 2.0) | |
| | Range | (0.0; 70.0) | (0.0; 3.0) | | (0.0; 95.0) | (0.0; 10.0) | |

**Supplementary Table 6**: Association of nodal status with different immune parameters according the BC subtypes. P values in bold fall below the 0.05 significance level.

| | | TNBC | | | | | | | HER2+ | | | | | | |
|---|---|---|---|---|---|---|---|---|---|---|---|---|---|---|---|
| | | | | | | Pairwise comparisons | | | | | | | Pairwise comparisons | | |
| **Variable** | **Statistic** | **pN0** | **pN1** | **pN2-3** | **P-value** | **1 vs 2** | **1 vs 3** | **2 vs 3** | **pN0** | **pN1** | **pN2-3** | **P-value** | **1 vs 2** | **1 vs 3** | **2 vs 3** |
| | **N** | **61** | **43** | **6** | | | | | **18** | **20** | **15** | | | | |
| **TILs** | Mean | 22.5 | 19.2 | 20.8 | 0.728 | 0.995 | 0.521 | 0.355 | 21.1 | 14.7 | 14.0 | 0.948 | 0.784 | 0.807 | 0.944 |
| | Std | 21.8 | 18.2 | 11.1 | | | | | 21.9 | 13.0 | 13.8 | | | | |
| | Median | 10.0 | 10.0 | 20.0 | | | | | 10.0 | 10.0 | 10.0 | | | | |
| | IQR | (5.0; 40.0) | (5.0; 20.0) | (20.0; 20.0) | | | | | (5.0; 30.0) | (5.0; 20.0) | (5.0; 20.0) | | | | |
| | Range | (5.0; 70.0) | (5.0; 80.0) | (5.0; 40.0) | | | | | (5.0; 70.0) | (5.0; 50.0) | (5.0; 60.0) | | | | |
| **PD1** | Mean | 6.1 | 7.56 | 5.0 | 0.355 | 0.278 | 0.423 | 0.296 | 7.8 | 5.2 | 8.7 | 0.062 | 0.046 | 1000 | 0.038 |
| | Std | 3.9 | 6.111 | 0.0 | | | | | 5.2 | 4.1 | 7.2 | | | | |
| | Median | 5.0 | 5.0 | 5.0 | | | | | 5.0 | 5.0 | 5.0 | | | | |
| | IQR | (5.0; 5.0) | (5.0; 10.0) | (5.0; 5.0) | | | | | (5.0; 10.0) | (5.0; 5.0) | (5.0; 10.0) | | | | |
| | Range | (0.0; 30.0) | (0.0; 30.0) | (5.0; 5.0) | | | | | (0.0; 20.0) | (0.0; 20.0) | (5.0; 30.0) | | | | |
| **CD73** | Mean | 2.5 | 5.0 | 10.0 | **0.004** | 0.001 | 0.795 | 0.183 | 1.9 | 11.7 | 3.7 | 0.487 | 0.323 | 0.281 | 1000 |
| | Std | 7.2 | 7.1 | 24.5 | | | | | 3.0 | 27.2 | 5.2 | | | | |
| | Median | 0.0 | 5.0 | 0.0 | | | | | 0.0 | 0.0 | 5.0 | | | | |
| | IQR | (0.0; 5.0) | (0.0; 5.0) | (0.0; 0.0) | | | | | (0.0; 5.0) | (0.0; 5.0) | (0.0; 5.0) | | | | |
| | Range | (0.0; 50.0) | (0.0; 40.0) | (0.0; 60.0) | | | | | (0.0; 10.0) | (0.0; 90.0) | (0.0; 20.0) | | | | |
| **CD8** | Mean | 8.6 | 10.5 | 10.0 | 0.420 | 0.409 | 0.234 | 0.502 | 7.8 | 7.2 | 8.0 | 0.901 | 0.865 | 0.823 | 0.659 |
| | Std | 6.6 | 9.5 | 5.5 | | | | | 6.2 | 4.7 | 6.5 | | | | |
| | Median | 5.0 | 5.0 | 10.0 | | | | | 5.0 | 5.0 | 5.0 | | | | |
| | IQR | (5.0; 10.0) | (5.0; 10.0) | (5.0; 10.0) | | | | | (5.0; 10.0) | (5.0; 7.5) | (5.0; 10.0) | | | | |
| | Range | (5.0; 40.0) | (5.0; 50.0) | (5.0; 20.0) | | | | | (5.0; 30.0) | (5.0; 20.0) | (5.0; 30.0) | | | | |
| **CD3** | Mean | 15.3 | 15.6 | 16.7 | 0.667 | 0.463 | 0.529 | 0.751 | 13.6 | 13.2 | 13.0 | 0.872 | 0.719 | 0.923 | 0.631 |
| | Std | 16.0 | 14.0 | 13.3 | | | | | 15.6 | 11.8 | 13.9 | | | | |
| | Median | 10.0 | 10.0 | 15.0 | | | | | 10.0 | 5.0 | 10.0 | | | | |
| | IQR | (5.0; 20.0) | (5.0; 20.0) | (5.0; 20.0) | | | | | (5.0; 10.0) | (5.0; 25.0) | (5.0; 10.0) | | | | |
| | Range | (5.0; 70.0) | (5.0; 60.0) | (5.0; 40.0) | | | | | (5.0; 70.0) | (5.0; 40.0) | (5.0; 60.0) | | | | |
| **Ki67** | Mean | 65.9 | 71.2 | 65.8 | 0.583 | 0.308 | 0.707 | 0.988 | 53.6 | 52.7 | 58.3 | 0.779 | 0.826 | 0.703 | 0.472 |
| | Std | 23.5 | 20.8 | 32.1 | | | | | 25.2 | 22.7 | 25.7 | | | | |

| | | | | | | | | | | | | | | | |
|---|---|---|---|---|---|---|---|---|---|---|---|---|---|---|---|
| | Median | 70.0 | 75.0 | 80.0 | | | | | 52.5 | 55.0 | 70.0 | | | | |
| | IQR | (60.0; 85.0) | (60.0; 90.0) | (45.0; 85.0) | | | | | (30.0; 75.0) | (32.5; 72.5) | (30.0; 80.0) | | | | |
| | Range | (10.0; 95.0) | (5.0; 95.0) | (10.0; 95.0) | | | | | (10.0; 90.0) | (20.0; 85.0) | (15.0; 95.0) | | | | |
| **CD4** | Mean | 5.9 | 6.6 | 9.2 | 0.090 | 0.518 | 0.027 | 0.103 | 6.1 | 6.2 | 6.0 | 0.565 | 0.519 | 0.717 | 0.337 |
| | Std | 2.5 | 3.9 | 5.8 | | | | | 3.7 | 2.7 | 3.9 | | | | |
| | Median | 5.0 | 5.0 | 7.50 | | | | | 5.0 | 5.0 | 5.0 | | | | |
| | IQR | (5.0; 5.0) | (5.0; 5.0) | (5.0; 10.0) | | | | | (5.0; 5.0) | (5.0; 5.0) | (5.0; 5.0) | | | | |
| | Range | (5.0; 20.0) | (5.0; 20.0) | (5.0; 20.0) | | | | | (5.0; 20.0) | (5.0; 15.0) | (5.0; 20.0) | | | | |
| **CD68** | Mean | 17.4 | 14.6 | 12.5 | 0.542 | 0.292 | 0.617 | 0.900 | 9.7 | 12.0 | 11.0 | 0.817 | 0.697 | 0.539 | 0.833 |
| | Std | 14.5 | 11.0 | 4.2 | | | | | 6.3 | 10.4 | 6.9 | | | | |
| | Median | 10.0 | 10.0 | 10.0 | | | | | 10.0 | 10.0 | 10.0 | | | | |
| | IQR | (10.0; 20.0) | (5.0; 20.0) | (10.0; 15.0) | | | | | (5.0; 10.0) | (5.0; 12.5) | (5.0; 15.0) | | | | |
| | Range | (5.0; 80.0) | (5.0; 60.0) | (10.0; 20.0) | | | | | (5.0; 30.0) | (5.0; 40.0) | (5.0; 30.0) | | | | |
| **FoxP3** | Mean | 2.6 | 2.1 | 2.3 | 0.352 | 0.195 | 0.395 | 0.757 | 2.0 | 1.7 | 1.4 | 0.895 | 0.653 | 0.916 | 0.793 |
| | Std | 2.1 | 1.8 | 2.1 | | | | | 1.7 | 1.5 | 0.5 | | | | |
| | Median | 2.0 | 2.0 | 1.0 | | | | | 1.0 | 1.0 | 1.0 | | | | |
| | IQR | (1.0; 5.0) | (1.0; 2.0) | (1.0; 5.0) | | | | | (1.0; 2.0) | (1.0; 2.0) | (1.0; 2.0) | | | | |
| | Range | (1.0; 10.0) | (0.0; 10.0) | (1.0; 5.0) | | | | | (1.0; 5.0) | (0.0; 5.0) | (1.0; 2.0) | | | | |
| **PDL1** | Mean | 5.8 | 2.9 | 0.0 | 0.077 | 0.057 | 0.181 | 0.460 | 3.6 | 3.5 | 7.1 | 0.422 | 0.232 | 0.238 | 0.962 |
| | Std | 15.9 | 11.2 | 0.0 | | | | | 15.3 | 14.5 | 24.5 | | | | |
| | Median | 0.0 | 0.0 | 0.0 | | | | | 0.0 | 0.0 | 0.0 | | | | |
| | IQR | (0.0; 0.0) | (0.0; 0.0) | (0.0; 0.0) | | | | | (0.0; 0.0) | (0.0; 0.0) | (0.0; 0.0) | | | | |
| | Range | (0.0; 70.0) | (0.0; 60.0) | (0.0; 0.0) | | | | | (0.0; 65.0) | (0.0; 65.0) | (0.0; 95.0) | | | | |

**Supplementary Table 7**: Clinico pathological variable in the METABRIC cohort according the BC subtypes. P values in bold fall below the 0.05 significance level.

| | | HER2+ | TNBC | P-value |
|---|---|---|---|---|
| **N** | | **134** | **320** | |
| **Age (continuous)** | Mean | 53.1 | 55.6 | 0.052 |
| | Std | 11.7 | 13.6 | |
| | Median | 53.3 | 55.6 | |
| | IQR | (44.5-60.4) | (45.3-66.0) | |
| | Range | (22.0-90.1) | (26.7-96.3) | |
| **Age (category)** | ≤ 45 | 36 (26.9) | 79 (24.7) | 0.351 |
| | ≤ 70 | 86 (64.2) | 197 (61.6) | |
| | > 70 | 12 (9.0) | 44 (13.8) | |
| **Inferred Menopausal status** | Pre-menopausal | 54 (40.3) | 113 (35.3) | 0.338 |
| | Post-menopausal | 80 (59.7) | 207 (64.7) | |
| **Tumor grade** | 1 | 0 (0.0) | 4 (1.3) | 0.397 |
| | 2 | 20 (15.9) | 40 (12.9) | |
| | 3 | 106 (84.1) | 266 (85.8) | |
| | Missing | 8 | 10 | |
| **pT** | NA | | | |
| **pN** | NA | | | |
| **Nodal status** | negative | 48 (37.8) | 144 (48.2) | 0.056 |
| | positive | 79 (62.2) | 155 (51.8) | |
| **BMI (continuous)** | NA | | | |
| **BMI (category)** | NA | | | |
