# Supplementary material for: Comparison of the tumor immune microenvironment of primary hormone receptor-negative HER2-positive and triple negative breast cancer
Source: NPJ Breast Cancer. 2021 Sep 23;7:128. doi: 10.1038/s41523-021-00332-7 (PMC8460670; doi:10.1038/s41523-021-00332-7)
Supplement: Supplementary file 2 — Reporting Summary [file 41523_2021_332_MOESM2_ESM.pdf]

nature portfolio

Corresponding author(s): Vos Hanne

Last updated by author(s): Jun 15, 2021

Reporting Summary

Nature Portfolio wishes to improve the reproducibility of the work that we publish. This form provides structure for consistency and transparency in reporting. For further information on Nature Portfolio policies, see our Editorial Policies and the Editorial Policy Checklist.

Statistics

For all statistical analyses, confirm that the following items are present in the figure legend, table legend, main text, or Methods section.

n/a	Confirmed	
☐	☒	The exact sample size (*n*) for each experimental group/condition, given as a discrete number and unit of measurement
☐	☒	A statement on whether measurements were taken from distinct samples or whether the same sample was measured repeatedly
☐	☒	The statistical test(s) used AND whether they are one- or two-sided<br>*Only common tests should be described solely by name; describe more complex techniques in the Methods section.*
☐	☒	A description of all covariates tested
☐	☒	A description of any assumptions or corrections, such as tests of normality and adjustment for multiple comparisons
☐	☒	A full description of the statistical parameters including central tendency (e.g. means) or other basic estimates (e.g. regression coefficient) AND variation (e.g. standard deviation) or associated estimates of uncertainty (e.g. confidence intervals)
☐	☒	For null hypothesis testing, the test statistic (e.g. *F*, *t*, *r*) with confidence intervals, effect sizes, degrees of freedom and *P* value noted<br>*Give P values as exact values whenever suitable.*
☒	☐	For Bayesian analysis, information on the choice of priors and Markov chain Monte Carlo settings
☐	☒	For hierarchical and complex designs, identification of the appropriate level for tests and full reporting of outcomes
☒	☐	Estimates of effect sizes (e.g. Cohen's *d*, Pearson's *r*), indicating how they were calculated

Our web collection on statistics for biologists contains articles on many of the points above.

Software and code

Policy information about availability of computer code

Data collection	The METABRIC dataset analysed during the current study is available in the cbioportal repository, https://www.cbioportal.org/study/summary?id=brca_metabric.
Data analysis	SAS software (version 9.4 of the SAS System for Windows) and R 3.5.2. was used. METABRIC transcriptomic data were processed through CIBERSORT16 and EPIC25 software in their online versions.

For manuscripts utilizing custom algorithms or software that are central to the research but not yet described in published literature, software must be made available to editors and reviewers. We strongly encourage code deposition in a community repository (e.g. GitHub). See the Nature Portfolio guidelines for submitting code & software for further information.

Data

Policy information about availability of data

All manuscripts must include a data availability statement. This statement should provide the following information, where applicable:

- Accession codes, unique identifiers, or web links for publicly available datasets
- A description of any restrictions on data availability
- For clinical datasets or third party data, please ensure that the statement adheres to our policy

The METABRIC dataset analysed during the current study is available in the cbioportal repository, https://www.cbioportal.org/study/summary?id=brca_metabric. The H&E and immunohistochemistry datasets generated and analyzed during the current study are not publicly available but will be made available upon reasonable request, following ethics committee approval and a data transfer agreement, to guarantee the General Data Protection Regulation.

Field-specific reporting

Please select the one below that is the best fit for your research. If you are not sure, read the appropriate sections before making your selection.

- [x] Life sciences
- [ ] Behavioural & social sciences
- [ ] Ecological, evolutionary & environmental sciences

For a reference copy of the document with all sections, see nature.com/documents/nr-reporting-summary-flat.pdf

Life sciences study design

All studies must disclose on these points even when the disclosure is negative.

Sample size	No sample size was calculated. We included all patients who underwent upfront surgery at the University Hospitals Leuven (UHL) between 2005 and 2010. Patients were retrospectively selected from the prospectively collected database of the Multidisciplinary Breast Center at the UHL.
Data exclusions	No data were excluded.
Replication	Immunohistochemical stainings were not replicated due to the scarcity of tumor tissue from patients.
Randomization	No randomization, retrospective study
Blinding	No blinding, retrospective study

Reporting for specific materials, systems and methods

We require information from authors about some types of materials, experimental systems and methods used in many studies. Here, indicate whether each material, system or method listed is relevant to your study. If you are not sure if a list item applies to your research, read the appropriate section before selecting a response.

Materials & experimental systems

n/a	Involved in the study	
[ ]	[x]	Antibodies
[x]	[ ]	Eukaryotic cell lines
[x]	[ ]	Palaeontology and archaeology
[x]	[ ]	Animals and other organisms
[x]	[ ]	Human research participants
[ ]	[x]	Clinical data
[x]	[ ]	Dual use research of concern

Methods

n/a	Involved in the study	
[x]	[ ]	ChIP-seq
[x]	[ ]	Flow cytometry
[x]	[ ]	MRI-based neuroimaging

Antibodies

Antibodies used	A polyclonal CD3 antibody (IR50361-2, Agilent) and monoclonal antibodies were used for CD4 (clone 4B12, Agilent), CD8 (clone C8/144B, Agilent), CD68 (clone KP1, Agilent), CD73 (clone 1D7, Abcam), FoxP3 (clone 22510, Abcam), Ki67 (clone MIB-1, Agilent) and PD1 (clone NAT105, Abcam). PD-L1 (Dako-Agilent 22C3 antibody)
Validation	PD-1 was performed by the clinical pathology lab of UHL, which stricly adheres to the indication of the college of American Pathologists concerning the verification and validation of antibodies used diagnostic purposes and clinical decision making (Fitzgibbons PL et al Arch.Pathol. Lab. Med. 2014; 138: 1432-1443). The different antibodies were validated by using a series of different cases of tonsil or appendix to demonstrate consistent and reproducible pattern of staining in the germinal centers of the lymphoid structures. PD-L1 staining was centrally performed by Discovery Life Sciences (formerly QualTek Molecular Laboratories). This staining was validated as an immunohistochemistry assay for PD-L1 (Programmed Cell Death 1 Ligand, CD274, B7-H1) using the Merck & Co antibody clone 22C3 on FFPE tissues (Marisa Dolled-Filhart et al. Arch Pathol Lab Med 1 November 2016; 140 (11): 1259–1266. doi: https://doi.org/10.5858/arpa.2015-0544-OA

Clinical data

Policy information about clinical studies

All manuscripts should comply with the ICMJE guidelines for publication of clinical research and a completed CONSORT checklist must be included with all submissions.

Clinical trial registration	No registration on clinicaltrial.gov since it was a retrospective study. The study protocol was approved by the local medical ethics committee of the UHL (S58910, 9 May 2016)

Study protocol

No study protocol avalaible since it was a retrospective study.

Data collection

A consecutive series of female patients with a non-special type ER/PR negative invasive adenocarcinoma of at least 2 cm diameter were included. All patients underwent upfront surgery at the University Hospitals Leuven (UHL) between 2005 and 2010. Patients were retrospectively selected from the prospectively collected database of the Multidisciplinary Breast Center at the UHL. Patients who received neo-adjuvant systemic treatment were excluded. Clinico-pathological parameters of all patients were retrieved from the hospital information system. BMI categories were defined as lean (<25 kg/m2) versus overweight (≥25 and <30 kg/m2) and obese (≥30 kg/m2).

Outcomes

Retrospective study, no outcomes were defined.
